# Five-Year Trend Analysis of Malaria Cases in East Shawa Zone, Ethiopia

**DOI:** 10.4314/ejhs.v31i6.17

**Published:** 2021-11

**Authors:** Temesgen File, Bayissa Chala

**Affiliations:** 1,2 Department of Applied Biology, School of applied Natural Sciences, Adama Science and Technology University. P.O..Box. 1888, Adama, Ethiopia

**Keywords:** Malaria, Plasmodium falciparum, Plasmodium vivax, prevalence, slide positivity rate

## Abstract

**Background:**

Malaria is an infectious disease caused by Plasmodium parasites. Of the five human malaria parasites Plasmodium falciparum and Plasmodium vivax are the two co-endemic predominant and widely distributed species in Ethiopia, with major public health importance. Even though enormous effort has been made countrywide to reduce the disease burden little was reported about trends of malaria transmission in the several localities of malarious areas like East Shawa Zone, Ethiopia. Thus, the present study was aimed at assessing fiveyear (2016–2020) trends of malaria transmission at Adama, Boset and Lume districts of East Shawa Zone of Oromia Regional State, Ethiopia.

**Methods:**

Retrospective data was extracted from the central surveillance database of East Shawa Zone Health Office. The data collected was analyzed from September 2020 to December 2020 to examine trends of malaria epidemiology in three malarious districts in the Zone.

**Results:**

The results of the present study showed a remarkable decrease in slide positivity rate (SPR) from 16.3 to 1.4% from 2016 to 2018 in the areas. However, a recent slight increase of malaria SPR was observed. On the other hand, as age increases more male individuals were infected with malaria compared to female of similar age groups. Falciparum, vivax and mixed malaria infection accounted for 53%, 41% and 6% respectively.

**Conclusions:**

Even though, an overall reduction of malaria incidence was revealed in the study areas, an increase in malaria SPR was observed in 2019 and 2020. Such inconsistency in reduction of malaria cases in the study area demands due attention of health planners.

## Introduction

Malaria is a mosquito-borne disease caused by *Plasmodium* parasites with a major public health problem in sub-Saharan Africa including Ethiopia. Still, it has been among the top ten ranking infectious diseases in Ethiopia despite significant progresses made in its intervention, which geared toward elimination. According to WHO report released in December 2019, there were 228 million worldwide cases of malaria in 2018, compared to 231 million in 2017. Though the disease is declining globally, WHO African region continues to carry a disproportionately high share of the global malaria burden with more than 90% of malaria related morbidity and mortality (1).

Of the five species of *Plasmodium* parasites that cause human malaria, *Plasmodium falciparum* and *Plasmodium vivax* are by far the most predominant and widely distributed in Ethiopia accounting for 60% and 40% of malaria cases, respectively. The two species, namely: *P. falciparum* and *P. vivax* are exceptionally coendemic in the country, which also happens in other few African countries (2). *Anopheles arabiensis*, a member of the *Anopheles gambiae* complex, is the principal malaria vector in Ethiopia with a wide geographical distribution (1, 5).

Ethiopia is one of the malaria epidemic prone countries in Africa where malaria is prevalent in over 75% of the country's land mass. Over 68% of the total population resides in the area below 2000 m altitude, which is considered to be at risk of malaria (3, 4). Since 2004, as a result of the deployment of huge workforce of health extension workers to expand community-based malaria intervention, introduction of effective malaria treatment options, and rapid diagnostic test (RDT), coupled with effective vector control tools widespread malaria epidemics have not been largely reported, except small scale outbreaks and seasonal case buildups (5). However, malaria is remaining a major health problem in the country (6). The major factors that limited the national effort to combat malaria in Ethiopia are; the challenges related to effective implementation of major intervention strategies for malaria control like early diagnosis, prompt treatment, selective vector control, environmental management, and resource intensive nature of the programs (3). Consequently, Tadesse *et al.* (3) reported that malaria is the third leading cause of outpatient department (OPD) visits (36%) in the East Shawa Zone of Oromia Regional State in 2012.

Ethiopia has recently targeted malaria elimination nationwide in 2030, through intensifying the existing malaria control activities (4, 7). For the success of this strategic objective, analyzing the trends of malaria cases at local level is paramount to understand the dynamics of malaria transmission and the status of the effectiveness of malaria interventions targeted to curb the disease burden. Likewise, this study intended to examine the status of malaria cases in the study areas by analyzing five-year retrospective data from Adama, Boset, and Lume districts. Trend analysis of malaria transmission status is an input to malaria surveillance for targeted intervention as stipulated in the national malaria elimination roadmap by 2030 (5). The aim of this study is therefore, to assess the five-year trends of malaria cases and the pattern of its annual and inter-annual transmission over sex, age, and seasonal dynamics in the study area.

## Methods

**Figure 1:**
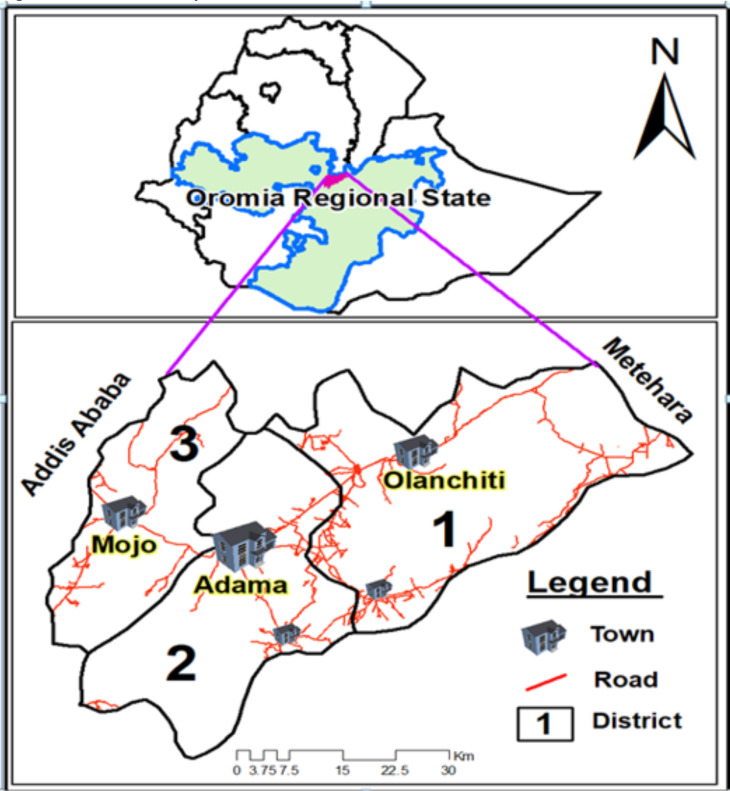
Physical location of Adama, Boset and Lume districts, (Figure developed by using Arc-GIS Desktop version 10.4).

Description of the study area: This study was conducted in three districts of East Shawa Zone administration of Oromia Regional State. The districts (Adama, Boset and Lume) are located surrounding Adama town, which is located at 8.54°N and 39.27°E, at about 99 km southeast of Addis Ababa. The surrounding districts were: Adama district, which includes the peri-urban, rural and smaller towns located around the Adama town administration. Lume district is located surrounding its own capital (Mojo town) found at the distance of 16 km northwest Adama town, and Boset district having its capital Olanchiti located at about 23 km northeast of Adama town. The districts selected for this study are located in East Shawa Zone surrounding Adama, the major town next to the capital in central Ethiopia. Due to warm temperature (16–32°C), rainfall patterns, and high humidity that is conducive for mosquito breeding the region is a malaria endemic area (8, 9). The areas are located within the Rift Valley area of central Ethiopia, having latitudinal location from 1436 - 1850m above sea level (Figure 1). Based on population projection of 2020 by Zonal health office of the districts had the total population of 392,922. In addition to its latitudinal location less than 2000m above sea level, heavy rainfall pattern during the summer season June to August, and a short rainy season around March creates favorable conditions for malaria transmission.

**Study design**: A retrospective study was conducted to analyze five years (2016–2020) trends of malaria cases in the study areas. The source of the data was microscopically and/or RDTs confirmed malaria cases as per the national parasitological diagnosis of malaria at health facilities and reported to zonal health office central surveillance through district health offices. Expertise of Health Information Management System (HIMS) managed the database, which consists of monthly total malaria cases by age and sex.

**Data analysis**: Retrospective data extracted and organized from a database of East Shawa zonal health office central surveillance was analyzed. The source of data for the database was respective district level surveillance report of malaria microscopy/RDTs results of confirmed malaria cases from each health facility. The data collected from district health facilities were organized and reported to zone health office central surveillance system. The database also consisted the total population projection in the districts during the study. Data on the total population, suspected malaria cases and people infected with malaria were collected, checked for completeness and consistency and double entered into Microsoft excel version 16 for analysis. The excel data was transported to graphic and data analysis software (ORIGINPRO^®^ version 9), to summarize and display the trends of malaria epidemiology in the area through table, bar graphs and line graphs.

**Ethical considerations:** The data were accessed after approval of the ethical clearance protocol of this study from Adama Science and Technology University certificate reference number RECSoANS/BIO/01/2019 and consent of Oromia Regional State Health Bureau.

## Results

**Figure 2:**
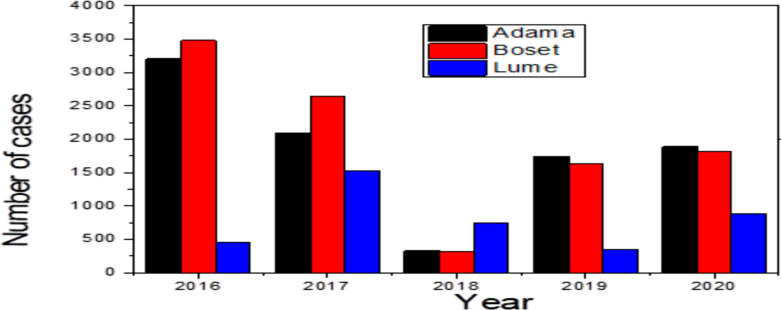
Five-year trends of malaria cases in Adama, Boset and Lume districts of East Shawa Zone, Oromia, Ethiopia, from 2016–2020.

Annual trends of malaria cases: From (2016 to 2020), 286,647 febrile-suspected cases were tested for malaria in the study area of which 23,110 (8.0%) were found malaria positive. The observed overall microscopic slide positivity rate showed a declining trend from 2016 to 2018, and slightly rose up in 2019 and 2020 as detailed in Table 1. Five year trends of malaria cases in the three districts showed higher cases detected in Boset 9893 (43%), followed by Adama 9252 (40%), and Lume 3965 (17%) districts. The overall trends of malaria cases decreased from 2016 to 2018. However, slight increase in the number of cases and SPR in 2019 and 2020. Moreover, the present study revealed temporal and spatial variation of malaria transmission in the three districts (Figure 2).

**Table 1:** Five-year annual trends of malaria cases in Adama, Boset and Lume districts of East Shawa Zone, Oromia, Ethiopia, from 2016–2020

Year	Total blood film examined	Total malaria positive cases	Slide positivity rate (SPR) (%)
**2016**	43,882	7,139	16.3
**2017**	58,105	6,259	10.8
**2018**	95,651	1,391	1.4
**2019**	43,879	3,729	8.4
**2020**	45,130	4,592	10.1

**Figure 3:**
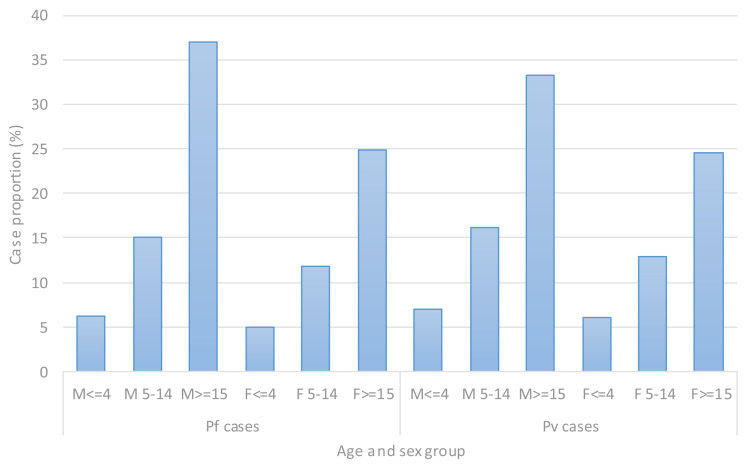
Yearly trends of confirmed malaria case proportion by age, sex and parasite species in Adama, Boset and Lume districts, East Shawa Zone, Oromia, Ethiopia, from 2016–2020.

**Trends of malaria cases by sex and age groups**: In children below the age of five, malaria case was similar in both sexes. However, as age increases more male individuals were infected with malaria compared to female of similar age groups, as detailed in Figure 3.

**Figure 4:**
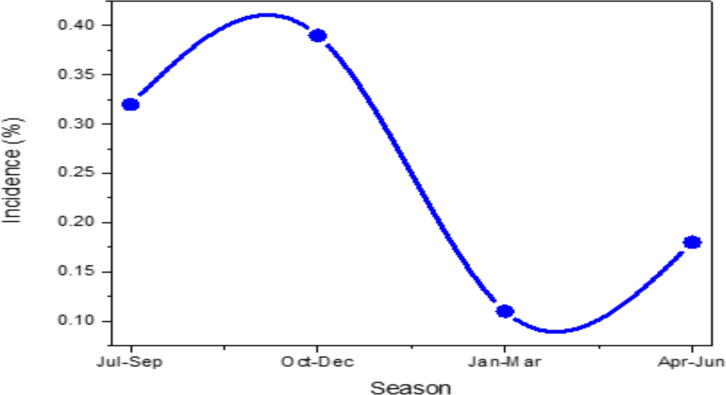
Retrospective data summary showing monthly trends of malaria cases in Adama, Boset and Lume districts, East Shawa Zone, Oromia, Ethiopia, from 2016–2020.

**Annual trends of malaria cases in different seasons of the year**: The five-year retrospective study showed, malaria was occurring throughout the year in the study areas. It showed seasonal, inter annual, and spatial variations. The major malaria peak season was in September and October, and the second minor peak was from May to June. The lowest malaria case was reported from January to March (Figure 4).

## Discussion

The present study revealed that, malaria is still remaining a major public health problem in the study areas. Five-year trend analysis indicated higher malaria cases in Boset followed by Adama and Lume districts. Although the overall malaria case has decreased from 2016 to 2018, SPR showed a slight increment in 2019 and 2020. During the study period, from 286,647 malaria suspected treatment seekers, 23,110 (8.0%) were microscopically/RDTs confirmed malaria cases (Table 1). These finding complements trend analysis study result in Wolkite; southwestern part of central Ethiopia (8.56%) (4), and higher than study conducted in Tigirai; northwestern of Ethiopia (6.96%) (10), lower than similar study conducted in western part of Ethiopia; Guba district of Benishangul Gumuz Zone (51.04%) (11), and cross-sectional study report from East Shawa Zone of Oromia Regional State in 2012 (25.2%) (3). Even though, the specific factors that accounted for such variation in malaria endemic regions need further investigation climatic factors and the status of malaria interventions in the region (10, 12) might have greatly contributed.

In the previous studies, it was reported that age-standardized malaria incidence and prevalence in Ethiopia between 1990 and 2015 showed a declining trend (13). However, in the present study maximum SPR was observed in 2016 (16.3%), and the minimum was in 2018 (1.4%). and slightly increased in 2019 and 2020 (Table 1 and Figure 2). The SPR in the study area, it is higher when compared malaria microscopic prevalence according to the national malaria indicator survey conducted in 2007, 2011, and 2016, which ranged from (0.5 to 1.3%) (14). The reduction of SPR was not consistent in the study area. In addition to the general suitable climate in the region, various epidemiological factors exist that favor mosquito breeding contributing for transmission heterogeneity of malaria in the study areas. The major factors that contributed to such inconsistency in maintaining progressive decline of malaria incidence demand further investigation. However, the present finding indicated the need for rigorous efforts in implementing the strategic actions stipulated in the national malaria elimination roadmap, which includes; effective surveillance system, quality assured diagnosis and treatment, targeted vector control measures, and introducing sensitive diagnostic tools like Polymerase Chain Reaction (PCR) (5).

With respect to age, consistent with the previous report (8–11, 15–20) the present study revealed that as age increases malaria incidence in males was higher than females of the same age group (Fig.3). The major factor that may account for higher malaria cases in male compared to female is that older boys and men may be at special risk for malaria from occupational and travel-related activities (5, 21).

Concerning malaria cases detected by species from the blood film, the present study revealed that out of 23,110 confirmed malaria cases 53%, 41% and 6% P. *falciparum, P. vivax* and mixed malaria infection, respectively. The current study finding is in agreement with the previous reports (10, 11, 22). The predominance of *P. falciparum* was consistent over the five years with slight seasonal variation. *P.vivax* cases during the minimal malaria season in the study area could be largely due to relapse.

Regarding seasonal transmission dynamics, year-round malaria cases were observed in the three districts of the study areas (Fig. 4). However, the highest peak was in the month of September and October (autumn), due to the formation of stagnant water and higher relative humidity suitable for mosquito breeding after the summer rainy season. This malaria peak season observed in the study areasis in agreement with similar studies conducted in different localities in Ethiopia (17, 22, 23). The second minor malaria season was during May to June (Spring) following short rainy season in the country, which is in agreement with the report of Gemechu *et al.* (16) and Sena *et al.* (19).

Determinants of such seasonal variation of malaria transmission in the study sites might be due to variation in seasonal temperature, topographic features, the condition of the residential areas (availability bushy gorges, shanty dwelling, untidiness, etc.) (9, 24) also, the economic factors of the inhabitant population are some of the common epidemiological factors for malaria incidence in the study areas.

Despite sharp decline in trends of malaria cases from 2016 to 2018, an increase in the trend was observed from 8.4% in 2019 to 10.1% in 2020. Such inconsistency in trends of malaria transmission in the study areas demand due attention by intensifying surveillance, quality assured early diagnosis and effective case management.
